# Adipose-Derived Stromal Cells Attenuate Adipose Inflammation in Obesity through Adipocyte Browning and Polarization of M2 Macrophages

**DOI:** 10.1155/2019/1731540

**Published:** 2019-12-04

**Authors:** Wen-Chao Zhang, Feng Qin, Xiao-Jun Wang, Zhi-Fei Liu, Lin Zhu, Ang Zeng, Ming-Zi Zhang, Nan-Ze Yu, Xiao Long

**Affiliations:** Division of Plastic and Reconstructive Surgery, Department of Plastic Surgery, Peking Union Medical College Hospital, Chinese Academy of Medical Sciences and Peking Union Medical College, Beijing 100032, China

## Abstract

Obesity is a metabolic condition associated with multiple health problems such as endocrine and metabolic dysfunction and chronic inflammation in adipose tissues. In this study, the ADSCs could be stimulated to differentiate into brown adipocyte with rosiglitazone treatment based on the Oil-Red-O staining trial. Furthermore, the multilocular lipid droplets located in the center was increased in differentiated brown adipocytes, and brown fat-associated proteins, UCP1, PPAR-*γ*, and LPL were highly expressed in brown adipocytes differentiated from ADSCs. Additionally, the results of animal experiments showed that both weight and amount of VLDL and LDL were decreased in the serum of obese mice after transplantation of rosiglitazone-induced brown adipocytes, while the level of HDL increased. Moreover, the proteins associated with lipid metabolism, LPA and UCP1, were downregulated, and the inflammatory response was suppressed through inhibition of the ITGAM/NF-*κ*B-mediated proinflammatory responses and polarization of M2 macrophages. Similarly, the amounts of proinflammatory cytokines, TNF-*α*, IL-6, and IL-1*β* were decreased after rosiglitazone-induced brown adipocyte transplantation. On the contrary, anti-inflammatory cytokine IL-10 was significantly increased in both groups of obese mice, with or without brown adipocyte transplantation. Therefore, the adipose-derived stromal cells with induced browning could promote lipid consumption and alternative polarization of M2 macrophages to attenuate adipose inflammation in obesity mouse models, which thus provides a potential therapy for obesity.

## 1. Introduction

Obesity is a disease accompanied by abnormal fat deposition and is closely associated with endocrine and metabolic dysfunction, inflammation, oxidative stress, and insulin resistance [1]. Obesity is a high-risk factor for type 2 diabetes, dyslipidemia, and cardiovascular diseases. Therefore, finding an effective and efficient approach to overcome obesity is very attractive for researchers, clinicians, and afflicted patients. In the adipose tissues of humans, there are three types of adipocytes, namely, white adipocytes, brown adipocytes, and beige adipocytes. White adipocytes mainly regulate lipid synthesis and storage, whereas both brown adipocytes and beige adipocytes significantly contribute to regulation of energy consumption through activation of uncoupling protein 1 (UCP1) in mitochondria [2, 3]. Additionally, both brown and beige adipocytes could produce a set of adipokines to engage in pathways for autocrinal, paracrinal, and metabolic activities [4]. For example, the TGF-*β* family produced by localized adipose tissues can contribute to the modulation of obesity [5] and can influence meteorin hormone-mediated immune-adipose interactions to promote beige fat thermogenesis [6]. To date, research on the different functions in metabolism that have important relationships to and can be influenced by brown and beige adipocytes has not been extensively undertaken [7].

Adipose-derived mesenchymal stromal cells (ADSCs) have multiple functions including cell renewal, spontaneous repair, and maintenance of homeostasis in adipose tissue [8, 9]. Further, several researchers have found that the ADSCs were able to differentiate into adipogenic cells [10, 11]. In addition, PPAR-*γ* agonists (i.e., rosiglitazone) have been shown to induce white adipocytes to becoming brown adipocytes [12]. Likewise, secreted IL-6 induced beige adipocytes to differentiate into products including brown adipocytes [13]. Some research indicates that adipocyte browning could promote lipolysis, thermogenesis, and insulin sensitivity and reduce circulating inflammatory response [14–16]. Therefore, ADSCs have great potential to control and be used as a treatment for obesity-associated inflammation and metabolic disorders with lineage differentiation resulting in browning of adipocytes as a means to help maintain homeostasis in microenvironments [17]. Thus, in our study, we attempted to identify characteristics of ADSCs undergoing induced browning of adipocytes as a means to develop a treatment-based approach to mediate obesity in animal subjects.

## 2. Materials and Methods

Samples came from 2-4-week-old C57Bl/6J mice (range of weights = 10-15 g), 5-6-week-old C57Bl/6J mice (15-20 g), and 5-6-week-old C57Bl/6J-ob/ob mice (30-35 g) and were sourced from SiBeiFu Laboratory Animal Technology Company, Beijing, China. Samples from dissections were placed into Dulbecco's modified Eagle medium (Hyclone), with additions of fetal bovine serum (Gibco), penicillin-streptomycin antibiotics, collagenase type I (Sigma), insulin (Sigma), rosiglitazone (Sigma-Aldrich), dexamethasone (Sigma-Aldrich), and isobutyl-methylxanthine (IBMX; Sigma-Aldrich). We used 24-well culture plates (Corning), 96-well culture plates (Corning), a high-fat diet (HFD), Oil-Red-O solution (abcam), paraformaldehyde (Sigma-Aldrich), isopropanol (Sigma-Aldrich), hematoxylin (Sigma), glycogelatin (Solarbio), anti-PPAR-*γ* (CST), anti-FABP4 (CST), anti-LPL (abcam), anti-UCP1 (CST), F4/80 antibody (BioLegend), CD206 antibody (BioLegend), SDS-PAGE assay products (beyotime), TNF-*α*, IL-6, IL-1*β*, IL-10 ELISA kits (MultiSciences), Cholesterol Assay Kit-HDL, LDL/VLDL (abcam), anti-LPA (abcam), anti-ITGAM (CST), and anti-NF-*κ*B (CST).

### 2.1. Bioinformatic Analysis

We downloaded data for mRNA expression from the GEO database (GSE71293) [18] and conducted a Gene Set Enrichment Analysis of mRNA expression profiles in every 4 hMADS samples, whereas the remaining 4 hMADS samples were dealt with rosiglitazone. The level of significance for differentially expressed genes at which the null hypothesis of no significant differences would be rejected was for *P* values < 0.05, │degrees of freedom│ > 4.

### 2.2. Isolation and Differentiated ADSCs

ADSCs were dissected from abdominal subcutaneous adipose tissues of the 2-4-week-old C57BL/6 mice. The mice were anesthetized and sacrificed, and we isolated the abdominal subcutaneous adipose tissues from the 2-4-week-old C57BL/6 mice. Afterwards, we washed collected adipose tissues extensively (three times) with PBS (pH = 7.45), then lysed the tissues in individual culture vessels with equal volumes of 1 mg/mL collagenase type I solution (1 mg/mL in PBS) for 60 min. Thereafter, we dissected out and washed the stromal vascular fraction, then transferred the cells and the medium from the culture vessel into new 15 mL sample tubes which we centrifuged at 6000 g/min for 12 minutes. Next, we isolated ADSCs by resuspension of the samples in DMEM containing 10% fetal bovine serum (FBS), insulin (10 *μ*g/mL), dexamethasone (concentration at 1 *μ*M), and IBMX (0.5 mM). Then, rosiglitazone (20 mg/mL) was added in the presence or absence of the medium to cultivate the ADSCs for 14 days. Finally, we refreshed the cultured medium and visualized morphology of ADSCs using inverted microscopy (LEICA) at a frequency of every two days until the ADSCs were found to have undergone a high degree of differentiation.

### 2.3. Oil-Red-O Staining

The well-differentiated ADSCs were stained with a 0.2% (*w*/*v*) Oil-Red-O solution for 20 min at room temperature after being fixed with 10% paraformaldehyde. Then, we washed the cells with isopropanol and dyed the nuclear regions with hematoxylin. Thereafter, we sealed the cells with glycogelatin and visualized the results with inverted microscopy (LEICA).

### 2.4. Animal Experimentation

The 5-6-week-old C57Bl/6J-ob/ob mice (*N* = 48) were randomized into four treatment groups, and the resultant male-female mice rations among groups did not significantly differ. All the mice were continually fed with a high-fat diet of a known composition (HFD, 60% Kcal of fat, Research Diets, NJ) for 1 week of adaptive cultivation, while the 5-6-week-old C57Bl/6J normal mice (*N* = 12) were fed with a normal diet and water for 1 week of adaptive cultivation (normal control group). Among the four groups of the C57Bl/6J-ob/ob mice, three groups of ob/ob mice were received and treated to induce browning of adipocytes with an injection (3 × 10^5^ cells/mL). Detailed protocols include inducing browning of adipocytes with the application of ADSCs from the 2-4-week-old C57BL/6 mice as was described above that were also suspended in DMEM. Then, 0.5 mL of the cell-cultured DMEM medium containing 3 × 10^5^ cells/mL was slowly injected into the abdominal adipose tissues of obese mice with a 26-G needle. Meanwhile, one group of these three groups of ob/ob mice having undergone the cell transplantation was divided and received an injection of 10 mg/kg rosiglitazone gavage. The other remaining groups of the ob/ob mice that underwent cell transplantation were divided into those that did or did not receive a sh-PPAR-*γ* adenovirus plasmid injection in the tail vein, at the dosage of 100 vp/mL. The adenovirus plasmid was constructed at HanBio, Co. Ltd., China. The other three groups of mice were separately gavaged with an equivalent volume of sterile PBS. The treatment periods were continued for 14 days. All mice were weighed (nearest gram) at 1, 4, 7, 11, and 14 days from the initial period when the ob/ob mice received cell transplantation. After the 14th day of treatment, all mice were sacrificed, and we collected blood samples, as well as the abdominal subcutaneous adipose tissues.

### 2.5. Chemiluminescent Assay for LDL, VLDL, and HDL

Supernatant for each sample was obtained from the blood samples of mice. Blood samples were centrifuged at 12000 g/min. We then analyzed production of LDL, VLDL, and HDL using standard chemiluminescent immunoassays.

### 2.6. Western Blot

The differentiated ADSCs in the plates were harvested and lysed in a RIPA buffer and were supplemented with protease inhibitor PMSF (Beyotime, China) for 30 mins on ice. In addition, we collected abdominal subcutaneous adipose tissues from the sacrificed mice and lysed them in type I collagenase and RIPA buffer supplemented with a protease inhibitor PMSF for 45 mins on ice. Then the protein concentration was divided and measured using BCA kits (Beyotime, China). Later, 25 *μ*g of total proteins in each hole was loaded into wells of a 10% agarose upper PAGE gel and 5% PAGE gel for separation using a standard SDS-PAGE reaction, then transferred from the gel to the PVDF membrane (Millipore, USA). We later blocked membranes with TBST in a 5% nonfat milk solution at room temperature for 1 hr. Afterward, the band of the induced cells was blocked with primary antibodies of proteins PPAR-*γ*, FABP4, LPL, and UCP1 at 4°C overnight, and the band of adipose tissues was blocked with primary antibodies of proteins LPA, UCP1, ITGAM, and NF-*κ*B at 4°C for 12 hrs. Thereafter, samples were separately replaced by second antibody anti-mouse IgG at room temperature for 1 hr. Finally, we visualized the resultant protein immunoreactive bands with the ECL detection instrument (Thermo Fisher Scientific) and the use of a chemiluminescent substrate.

### 2.7. Flow Cytometry

Upon collection of blood samples from sacrificed mice, we centrifuged samples at 12000 g · min^−1^, then obtained the supernatant. We stained products with PE-Cy5-conjugated monoclonal antibody to test against mouse F4/80 (BioLegend). We followed this by staining with the APC-conjugated monoclonal antibody to test against the mouse CD206 (BioLegend). We also used isotypic antibodies as a control, and the Fc block was stained before experiment initiation. Resultant cells were analyzed using Calibur FACS (Bioscience, BD, USA).

### 2.8. ELISA for the Production of Inflammatory Cytokines TNF-*α*, IL-6, IL-1*β*, and IL-10

Supernatants were obtained from blood samples of mice and centrifuged at a high speed. We then analyzed inflammatory cytokines of TNF-*α*, IL-1*β*, IL-6, and IL-10 with ELISA kits according to the manufacturer's instructions.

### 2.9. Statistical Analysis

Differences were evaluated using one-way ANOVA with SPSS, IBM Corp., Version 19.0. Variables were represented as mean ± standard error, and the level of significance was at the *P* value < 0.05 for which the null hypothesis would be rejected and differences considered significant. We used this data to produce plots with GraphPad Prism Software.

## 3. Results

### 3.1. Gene Set Enrichment Analysis

**Figure 1:**
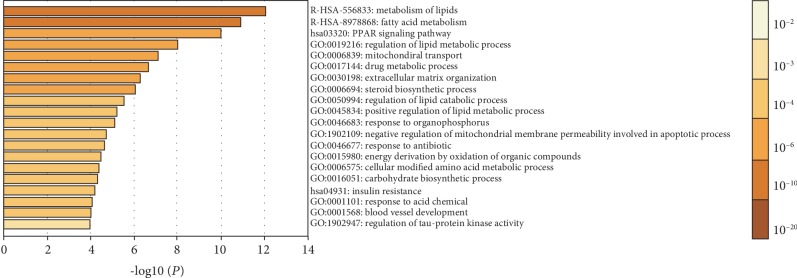
Biological process of GO ontology as well as the KEGG and REACTOME pathways related to the differentially expressed genes was analyzed using the R-based coding and DAVID tools. Major biological processes included lipid metabolism (GO:0019216), mitochondrial transport (GO:0006839), while the major signaling pathways included mainly the lipid metabolism pathway (R-HSA-556833 and R-HSA-8978868), PPAR-*γ* signaling pathway (hsa03320), and the insulin resistance pathway (hsa04931), among others.

The results indicated that the significant differentially expressed genes of the differentiated adipocytes engaged in lipid metabolism pathway, PPAR-*γ* signaling pathway, and insulin resistance pathway. Besides, the major biological processes of lipid metabolism and mitochondrial transport were found to be intertwined with the process of ADSC differentiation into adipocytes (Figure 1).

### 3.2. ADSCs Differentiated into White Adipocytes and Rosiglitazone Induced Adipocyte Browning

**Figure 2:**
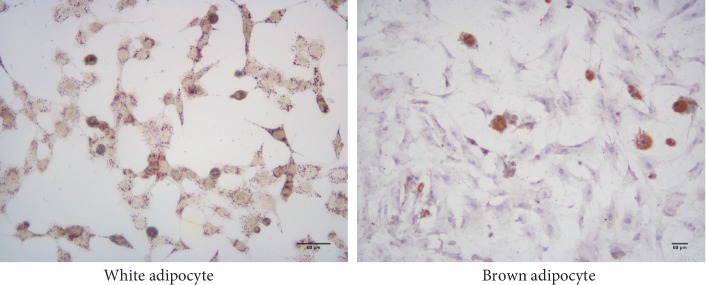
Large lipid droplets in the induced white adipocytes and the browning adipocytes with small lipid droplets were dyed using Oil-Red-O, and the location of the round nuclei located in the center was determined using microscopy at 200× magnification.

After 14 days of cultivation, the cells were stained with Oil-Red-O; the large lipid droplets accumulated in the white adipocytes were dyed; and while in the round nuclei of the browning adipocyte, the small lipid droplets were dyed (Figure 2).

### 3.3. Rosiglitazone Promoted Lipid Consumption and Induced Adipocyte Browning

**Figure 3:**
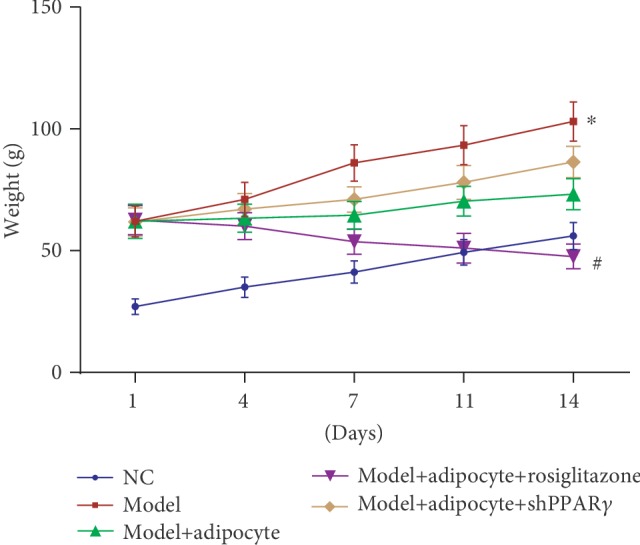
The weights of mice were evaluated during the treatment periods. # denotes *P* values < 0.05; weight differences were significant in the obese mice treatment group that received adipocyte transplantation combined with rosiglitazone treatment when compared to results for the obese mice treatment group without cell transplantation. ∗ denotes *P* values < 0.05; there were significant differences in the obese mice treatment group without cell transplantation compared with the normal control mice.

**Figure 4:**
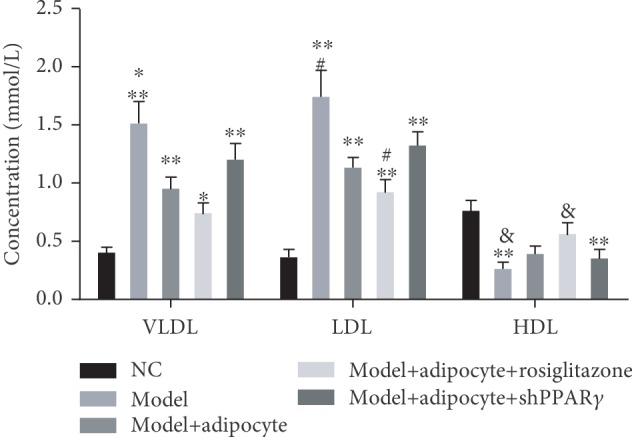
Intermediate metabolite lipids including VLDL, LDL, and HDL were analyzed using the cholesterol assay kit. ∗∗ denotes significant differences of VLDL in the obese mice treatment groups with or without cell transplantation except for the mice which received rosiglitazone compared with results for the normal control mice (*P* < 0.05). ∗∗ denotes the significant differences of LDL between the obese mice treatment group with or without cell transplantation compared with the normal control mice (*P* < 0.05). ∗∗ denotes the significant difference of HDL in the obese mice sample group without cell transplantation and in the mice that received adenovirus injections compared to results from the normal control mice (*P* < 0.05). ∗, #, and & all denote significant differences between VLDL, LDL, and HDL in the obese mice treatment group without cell transplantation compared with treatment group wherein the mice received rosiglitazone (*P* < 0.05).

**Figure 5:**
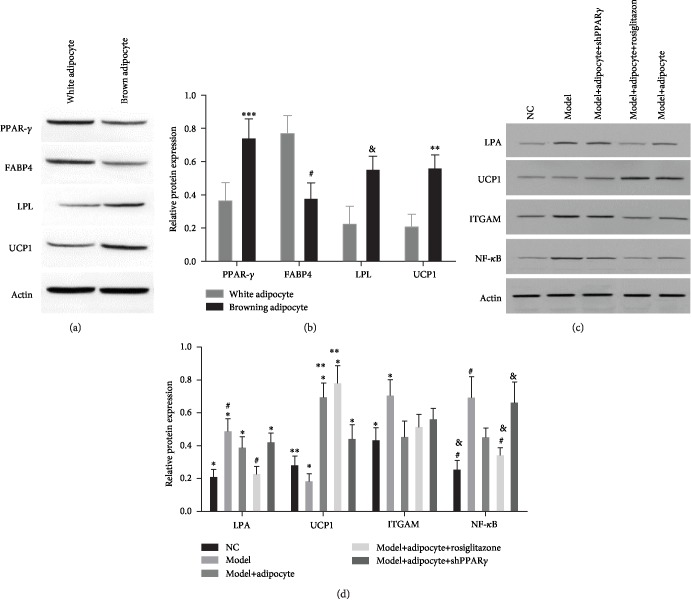
(a, c) Results from western blotting which analyzed the levels of expression of proteins. Proteins PPAR-*γ*, LPL, and UCP1 were highly expressed in the differentiated browning adipocytes, whereas the protein FABP4 was highly expressed in the cells induced to become white adipocytes. ∗∗∗, #, &, and ∗∗ all indicate that differences for the levels of protein expression were significant compared to results of the sample for which we induced white adipocytes (*P* < 0.05). (b) Proteins including LPA, ITGAM, and NF-*κ*B were upregulated in the obese mice treatment group compared with the normal control mice, whereas the expression of UCP1 was downregulated. ∗ indicates that there was a significant difference for the levels of protein LPA in the obese mice treatment group regardless of having cell transplantation except for the mice that received rosiglitazone for comparisons to the normal control mice (*P* < 0.05). # indicates that there was a significant difference in the levels of expression of LPA protein in the obese mice treatment group compared with the mice that received rosiglitazone (*P* < 0.05). ∗∗ indicates that there was a significant difference in the levels of expression of the protein UCP1 in the obese mice treatment group with cell transplantation and rosiglitazone treatment compared with the normal control mice (*P* < 0.05). ∗ indicates that there was a significant difference in the levels of the expression of the protein UCP1 in the obese mice treatment group with cell transplantation compared with the obese mice treatment group without cell transplantation (*P* < 0.05). ∗ indicates that there was a significant difference of the levels of the expression of the protein ITGAM in the obese mice treatment group without cell transplantation compared with the normal control mice (*P* < 0.05). # indicates that there was a significant difference in the levels of expression of the protein NF-*κ*B in the obese mice treatment group without cell transplantation compared with the normal control mice that also received rosiglitazone (*P* < 0.05). & indicates that there was a significant difference in the levels of the expression of the protein NF-*κ*B in the obese mice treatment group that also received adenovirus injections compared with the normal control mice and the mice that received rosiglitazone (*P* < 0.05; (d)).

After 14 days of treatment, there was variability in the weight of obese mice. Mice that received rosiglitazone along with an injection to induce browning of adipocytes lost a significant amount of weight compared to results for the treatment with the obese mice (*P* < 0.05, Figure 3). Additionally, obese mice without cell transplantation clearly had gained weight compared with the mice in other treatment groups (Figure 3). Further, lipids in known intermediate metabolites including VLDL and LDL were abundant in the obese mice treatment group without cell transplantation, and the amounts of VLDL and LDL were decreased in the mouse treatment group that received cell transplantation regardless of the presence or absence of rosiglitazone compared with the obese mice treatment group without cell transplantation. Conversely, the amount of HDL lipid metabolite was increased in the mice that underwent cell transplantation and in the presence or absence of rosiglitazone treatment compared with the obese mice without treatment. Further, the amounts of VLDL, LDL, and HDL in the normal control mice varied little (Figure 4). The proteins PPAR-*γ*, LPL, and UCP1 were highly expressed in the induced browning adipocytes, whereas the levels of expression of the protein FABP4 were higher in the differentiated white adipocytes (Figures 5(a) and 5(b)). Additionally, we found that the levels of expression in the abdominal subcutaneous adipose tissues of the obese mice of lipid-protein LPA and inflammatory-related protein ITGAM, NF-*κ*B, were both upregulated and that these characteristics were attenuated after the obese mice received browning adipocyte transplantation. Meanwhile, the PPAR-*γ* agonist rosiglitazone was able to continuously induce the browning of the transplanted adipose cells and suppressed the inflammatory response. The protein UCP1 and measures of mitochondrial energy metabolism were significantly upregulated, and the expression of protein LPA was decreased in the obese mice treatment group that received browning adipocyte transplantation. Additionally, the inflammatory response-related proteins ITGAM and NF-*κ*B were obviously downregulated compared with the obese mice treatment group without cell transplantation. Activities of lipid consumption pathways and anti-inflammatory responses were suppressed but only in a small manner after the obese mice received adenovirus injections compared to results from the mouse treatment group that received rosiglitazone (Figures 5(c) and 5(d)).

### 3.4. ADSC-Induced Browning of Adipocytes Contributed to Alternative Polarization of M2 Macrophages as a Means to Reduce Inflammation

**Figure 6:**
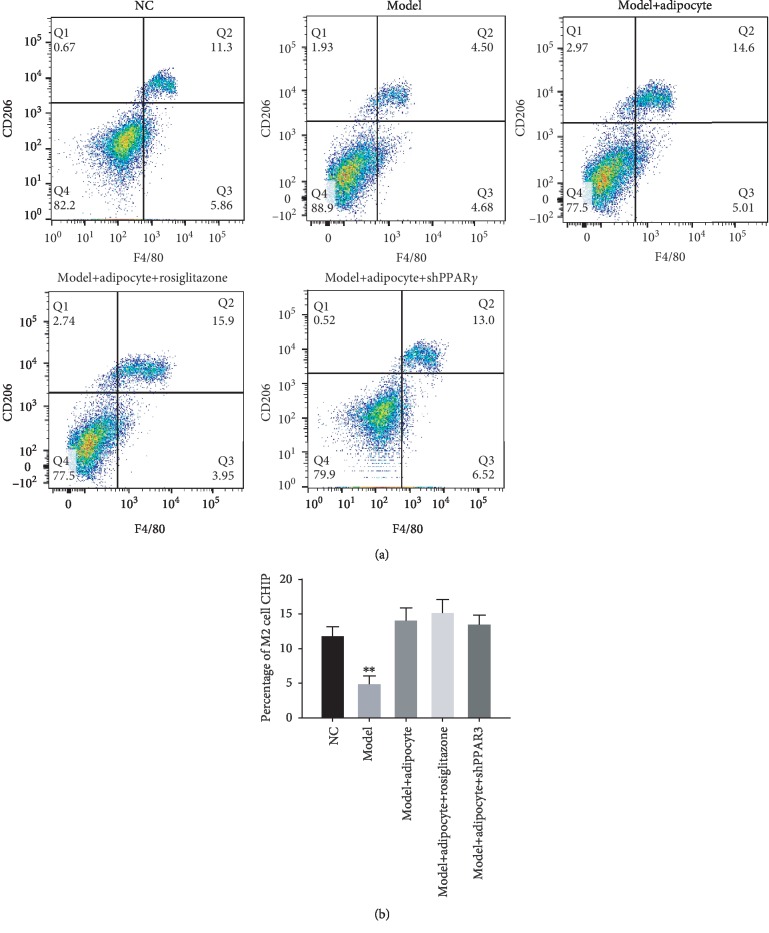
(a) Polarized M2 macrophages (F4/80+ and CD206+) were detected with fluorescence signals (Q2 bright field (upper right UR)). ∗∗ indicates that there was a significant difference of polarized M2 macrophages in the obese mice without cell transplantation compared with the normal control mice and the mice that received cell transplantation (*P* < 0.05; (b)).

**Figure 7:**
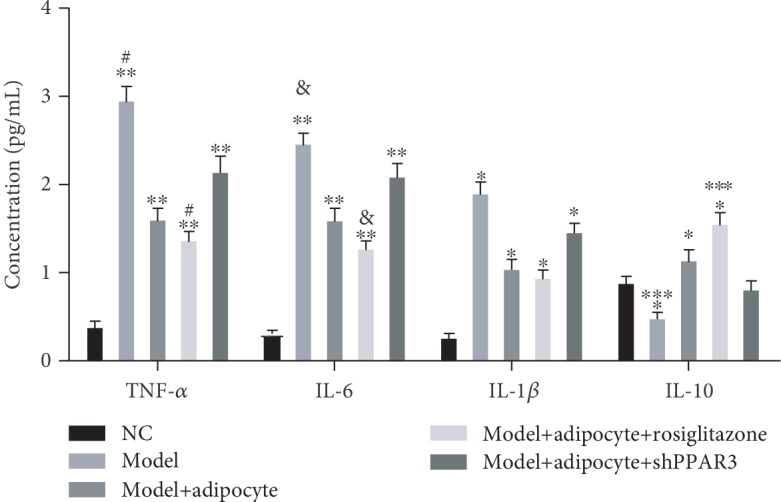
Secretion of TNF-*α*, IL-6, IL-1*β*, and IL-10 was examined by ELISA. Amounts of TNF-*α*, IL-6, and IL-1*β* were abundant in the obese mice. In contrast the production of IL-10 was increased after the obese mice received browning adipocyte transplantations and/or with the application of drug-based rosiglitazone treatments. ∗∗ indicates that there were significant differences for TNF-*α*, IL-6, and IL-1*β* concentrations in obese mice with or without cell transplantation compared with the normal control mice (*P* < 0.05). ∗ indicates that there were significant differences of IL-10 in obese mice with cell transplantation and rosiglitazone applications compared with obese mice without treatment (*P* < 0.05). #, &, and ∗∗∗ indicate significant differences in TNF-*α*, IL-6, and IL-10 concentrations in obese mice without treatment compared with the mice that received rosiglitazone (*P* < 0.05), respectively. Each data point is representative of at least three independent experiments that all had significant results.

Flow cytometry was used to analyze the macrophage phenotype. Results showed that the percent of polarized M2 macrophage stained with F4/80-positive and CD206-positive was increased after the mice received browning adipocyte transplantation. Further, treatment with rosiglitazone could enhance the effects of polarizing M2 macrophage (Figure 6), while the inflammatory cytokines TNF-*α*, IL-6, and IL-1*β* were abundant in the obese mice without treatment, and the production of TNF-*α*, IL-6, and IL-1*β* was alleviated after the mice received browning adipocyte transplantation and in the presence or absence of rosiglitazone. Conversely, the production of anti-inflammatory cytokine IL-10 was increased after mice received browning adipocyte transplantation and in the presence or absence of rosiglitazone compared to the obese mice without treatment (Figure 7).

## 4. Discussion

In our study, we explored the potential for overcoming obesity with the application of inducing browning of adipocytes by adipose-derived mesenchymal stromal cells. Our results indicated that the adipose-derived mesenchymal stromal cells (ADSCs) could differentiate into white and browning adipocytes with a high level of multipotency based upon the results of our study. Results for morphological characteristics of lipid droplets were different for comparisons of the induced adipocytes and the expression of the brown-specific gene UCP1. Likewise, the key regulator PPAR-*γ* influencing adipocyte differentiation was highly expressed in the induced browning adipocytes from the application of rosiglitazone. These findings are consistent with the conclusion of Petrovic [19].

Despite this, we did not identify whether or not the browning adipocytes were in actuality characteristically brown or beige. An important finding was that the beige adipocytes were recognized as the brown-like adipocytes derived from white adipocytes [20]. Additionally, brown and beige adipocytes have been identified to play important and distinct roles in repairing metabolic disorders and metabolic disease [21, 22]. Therefore, in our study, induction of browning adipocytes obviously made sense through transplantation into the constructed obese mice modeling efforts. Obese mice lost weight after having received the browning adipocyte transplantation. Further, the drug rosiglitazone enhanced such types of effects. We also found that the PPAR-*γ* agonist rosiglitazone contributed to promoting the induction and influenced the dynamics of the function of beige adipocytes [23]. Further, the induced browning of adipocytes has important ties in the pathways promoting energy expenditure and also appeared to promote triglyceride clearance and glucose disposal [24]. This thermogenic function was mainly mediated by UCP1 proteins located in the inner mitochondrial membrane of brown adipocytes, which in turn accelerated substrate oxidation of fatty acids [25]. Hence, data in our study showed that the amounts of VLDL and LDL were decreased after the obese mice received the browning adipocyte transplantation and that the levels of HDL production were increased. Similarly, these effects were enhanced with rosiglitazone treatment. Rosiglitazone is an agonist of PPAR-*γ*, and further, PPAR-*γ* is the master transcription factor of adipogenesis, which plays pivotal roles in the regulation of lipid metabolism, adipokine secretion, and insulin sensitivity [26, 27]. Therefore, rosiglitazone facilitated lipid metabolism and increased insulin sensitivity [27]. Correspondingly, the results of our study indicated that the levels of expression of apolipoprotein(a) were significantly decreased in the obese mice with rosiglitazone treatment. Additionally, the upregulation of the protein UCP1 in the samples of abdominal subcutaneous fatty tissue from obese mice showed that rosiglitazone continuously maintained the transplanted cells induced for browning. This finding was consistent with the conclusion of Li et al. [28]. Additionally, Siebert et al. [29] demonstrated that rosiglitazone had anti-inflammatory effects through its effect on PPAR-*γ* protein in adipocytes. Similarly, our results indicated that obese mice which received rosiglitazone treatment had significantly decreased levels of ITGAM and NF-*κ*B protein expression associated with inflammatory response and had decreased levels of polarized M2 macrophages, which ultimately suppressed the production of proinflammatory cytokines TNF-*α*, IL-6, and IL-1*β* and promoted the secretion of anti-inflammatory cytokine IL-10. Interestingly, adipocytes inducted to undergo browning also contributed to enhancing mitochondrial activity and insulin sensitivity and increased the numbers of anti-inflammatory M2 macrophages in the adipose bed [30]. Hence, the induction of browning of adipocytes through transplantation also played a key role in anti-inflammation pathways for obesity and lipid metabolism. Correspondingly, our study confirmed results for experimental treatments in which obese mice received cell transplantation in the absence of rosiglitazone treatment and in the presence of sh-PPAR-*γ* adenovirus plasmid injection in tail veins. With transfection of sh-PPAR-*γ* adenovirus plasmid in the obese mice treatment group, results indicated that the expression of UCP1 was downregulated, whereas the expression of ITGAM and NF-*κ*B proteins was upregulated. Further, proinflammatory responses were more evident with increased secretion of proinflammatory cytokines TNF-*α*, IL-6, and IL-1*β*, among others. Hence, administration of the sh-PPAR-*γ* adenovirus plasmid inhibited the ADSCs being induced to become browning adipocytes. Further, Saleh et al. [31] systematically analyzed the effects of AD-MSC transplantation on obesity and confirmed its promising application. Hence, there is great potential in that the adipose-derived mesenchymal stromal cells induced to be browning adipocytes may be a course of treatment for patients with obesity and would be achieved through cell transplantation.

## 5. Conclusions

In this study, we found that the ADSCs induced to become browning adipocytes contributed to promote lipid thermogenesis and the increased polarization of M2 macrophages as an adaptive response to reduce inflammation. Thus, our results provided a potential therapy for obesity.

## Data Availability

Access to this data will be considered by the author upon request.

## Abbreviations

ADSCs: Adipose-derived mesenchymal stromal cells
FABP4: Fatty acid-binding protein 4
ITGAM: Integrin alpha-M
LPA: Apolipoprotein(a)
LPL: Lipoprotein lipase
NF-*κ*B: Nuclear factor NF-kappa-B
PPAR-*γ*: Peroxisome proliferator-activated receptor gamma 2
UCP1: Uncoupling protein 1.

## Supplementary Materials

Supplementary Materials Supplementary file WB of Figure 5A: antibodies for western blot are as follows: anti-PPAR-*γ* (catalog: 95128S, CST), anti-FABP4 (catalog: 2120, CST), anti-LPL (catalog: ab137821, abcam), anti-UCP1 (catalog: 14670, CST). The groups from left to right are White adipocyte and Brown adipocyte. Supplementary file WB of Figure 5C: antibodies for western blot are as follows: anti-LPA (catalog: ab33470, abcam), anti-UCP1 (catalog: 14670, CST), anti-ITGAM (catalog: 46512, CST), anti-NF-*κ*B (catalog: 6956, CST). The groups from left to right are NC, Model, Model+Adipocyte+shPPAR*γ*, Model+Adipocyte+rosiglitazone, and Model+Adipocyte.
