# Corrigendum to “Adipose-Derived Stromal Cells Attenuate Adipose Inflammation in Obesity through Adipocyte Browning and Polarization of M2 Macrophages”

**DOI:** 10.1155/2022/9829413

**Published:** 2022-01-06

**Authors:** Wen-Chao Zhang, Feng Qin, Xiao-Jun Wang, Zhi-Fei Liu, Lin Zhu, Ang Zeng, Ming-Zi Zhang, Nan-Ze Yu, Xiao Long

**Affiliations:** Division of Plastic and Reconstructive Surgery, Department of Plastic Surgery, Peking Union Medical College Hospital, Chinese Academy of Medical Sciences and Peking Union Medical College, Beijing 100032, China

In the article titled “Adipose-Derived Stromal Cells Attenuate Adipose Inflammation in Obesity through Adipocyte Browning and Polarization of M2 Macrophages” [1], the authors identified error in Figure 5(a) which was introduced during the preparation of the manuscript. In Figure 5(a), the order of PPAR-*γ* protein bands in the WB results is reversed. The corrected Figure 5(a) is as follows:

## Figures and Tables

**Figure 1:**
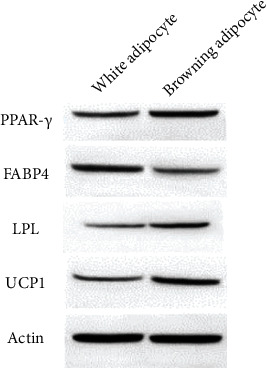
(a) Results from western blotting which analyzed the levels of expression of proteins. Proteins PPAR-*γ*, LPL, and UCP1 were highly expressed in the differentiated browning adipocytes, whereas the protein FABP4 was highly expressed in the cells induced to become white adipocytes.

## References

[1] Zhang W.-C., Qin F., Wang X.-J. (2019). Adipose-Derived Stromal Cells Attenuate Adipose Inflammation in Obesity through Adipocyte Browning and Polarization of M2 Macrophages. *Mediators of Inflammation*.

