# Supplementary material for: Adipose-Derived Stromal Cells Attenuate Adipose Inflammation in Obesity through Adipocyte Browning and Polarization of M2 Macrophages
Source: Mediators Inflamm. 2019 Dec 4;2019:1731540. doi: 10.1155/2019/1731540 (PMC6913309; doi:10.1155/2019/1731540)

Supplementary file WB of **Figure 5A**

Antibodies for western blot are as follows: anti-PPAR-γ (catalog: 95128S, CST), anti-FABP4 (catalog: 2120, CST), anti-LPL (catalog: ab137821, abcam), anti-UCP1(catalog: 14670, CST).

The groups from left to right are White adipocyte and Brown adipocyte.

Actin

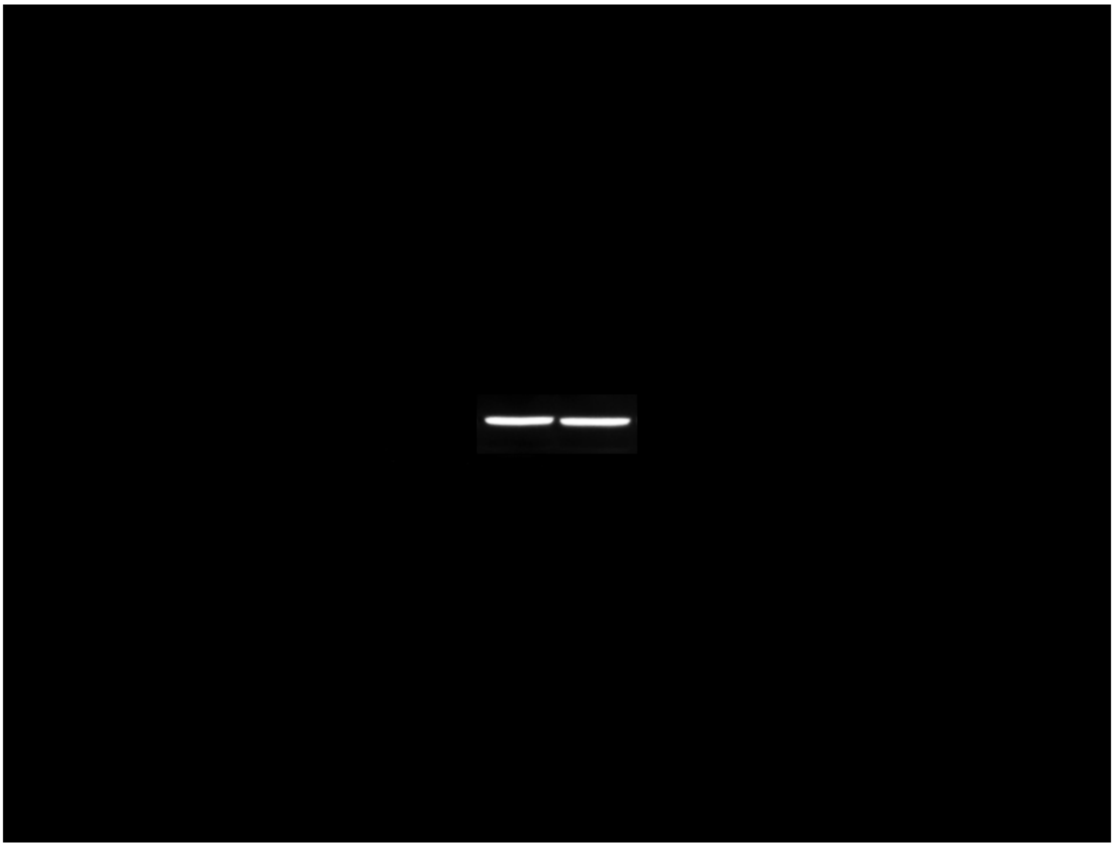

PPAR-γ

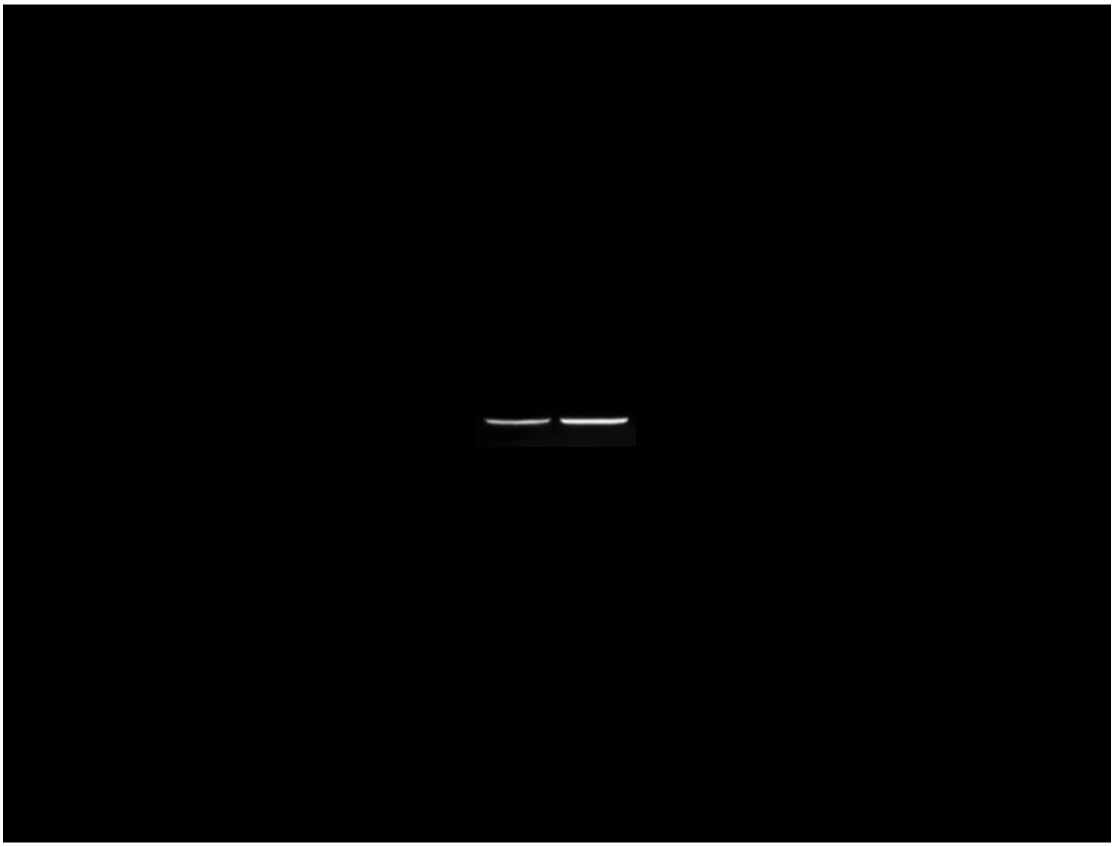

FABP4

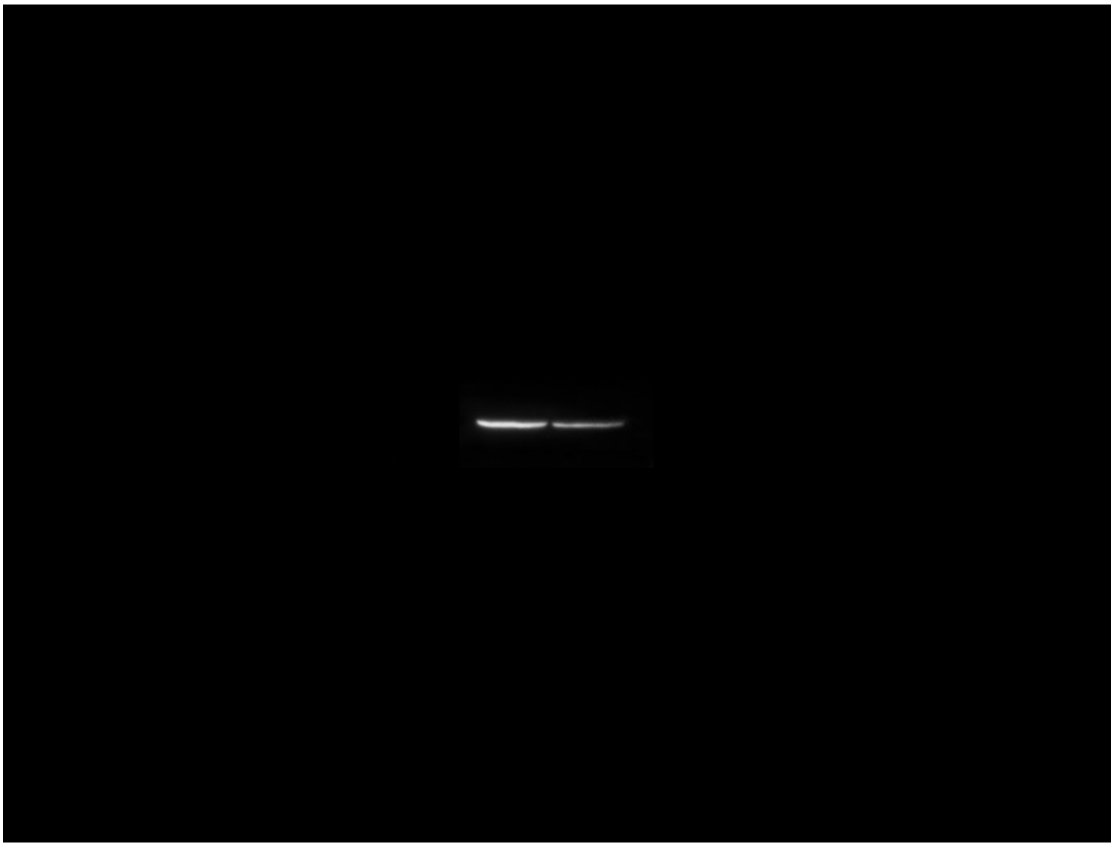

LPL

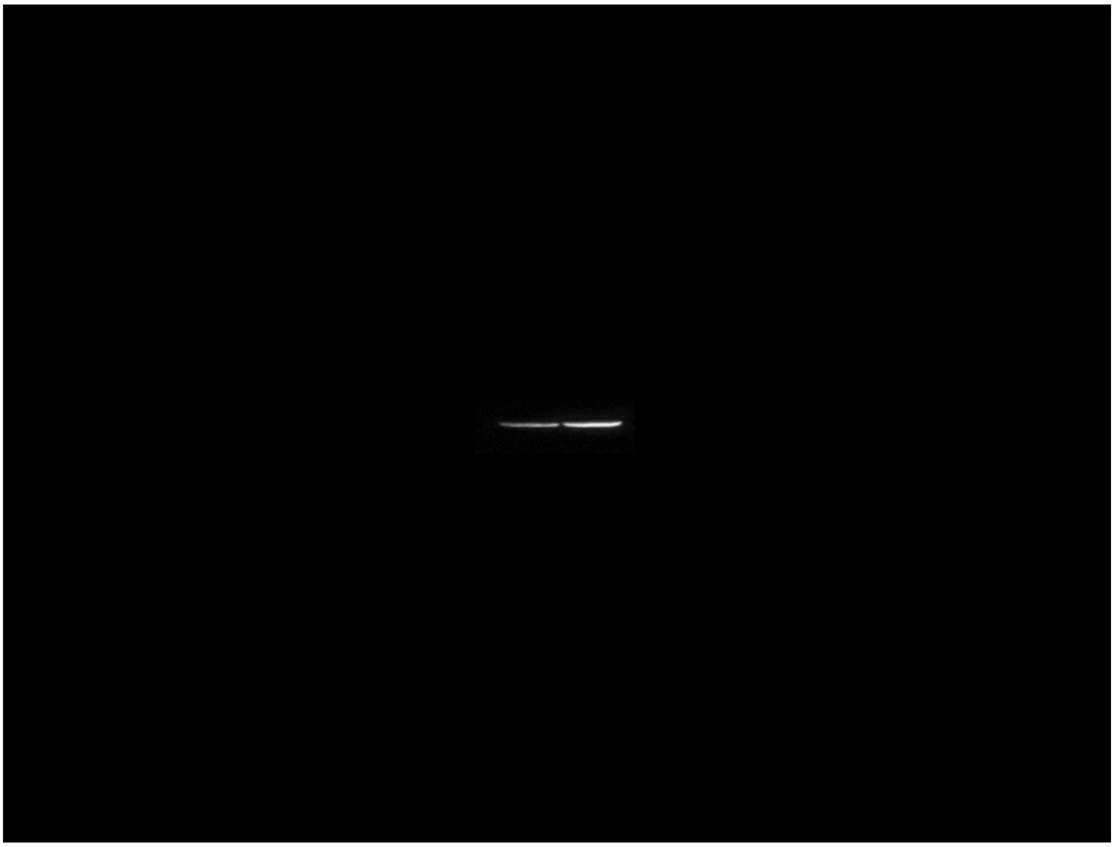

UCP1

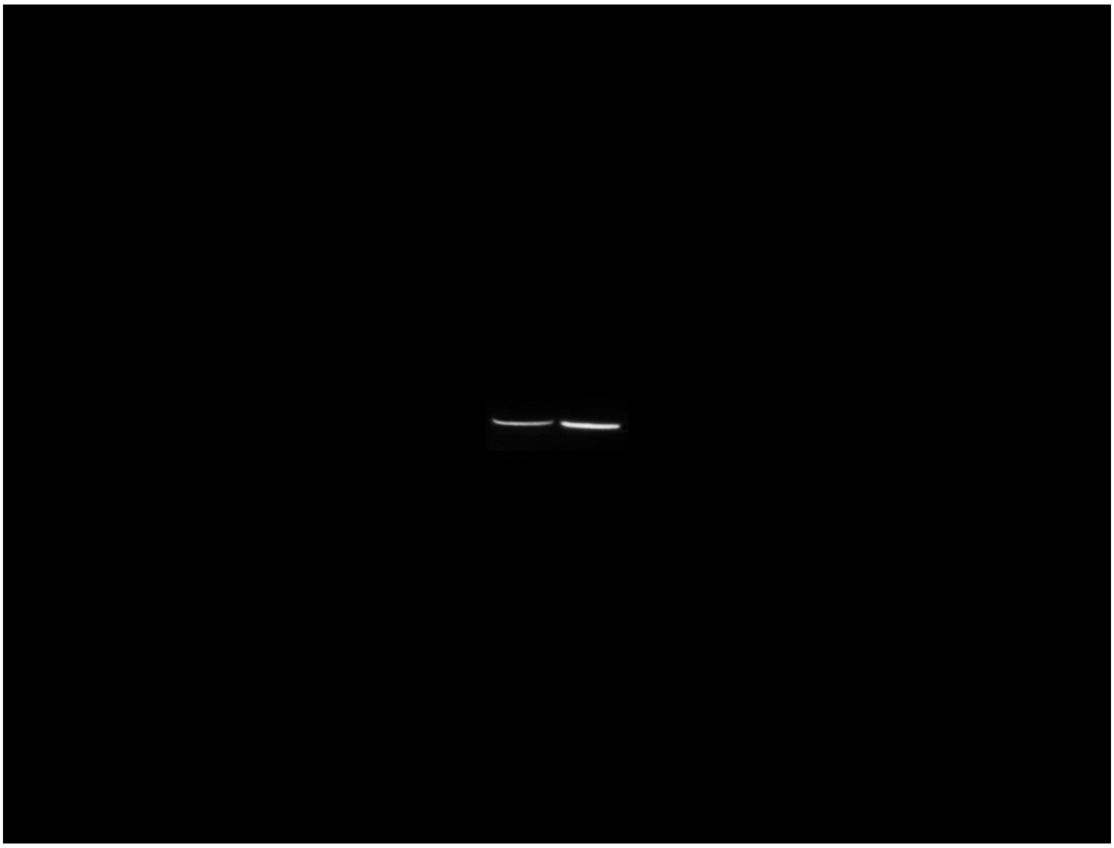

Supplementary file WB of **Figure 5C**

Antibodies for western blot are as follows: anti-LPA (catalog: ab33470, abcam), anti-UCP1 (catalog: 14670, CST), anti-ITGAM (catalog: 46512, CST), anti-NF-κB (catalog: 6956, CST).

The groups from left to right are NC, Model, Model+Adipocyte+shPPARγ, Model+Adipocyte+rosiglitazone, Model+Adipocyte.

Actin

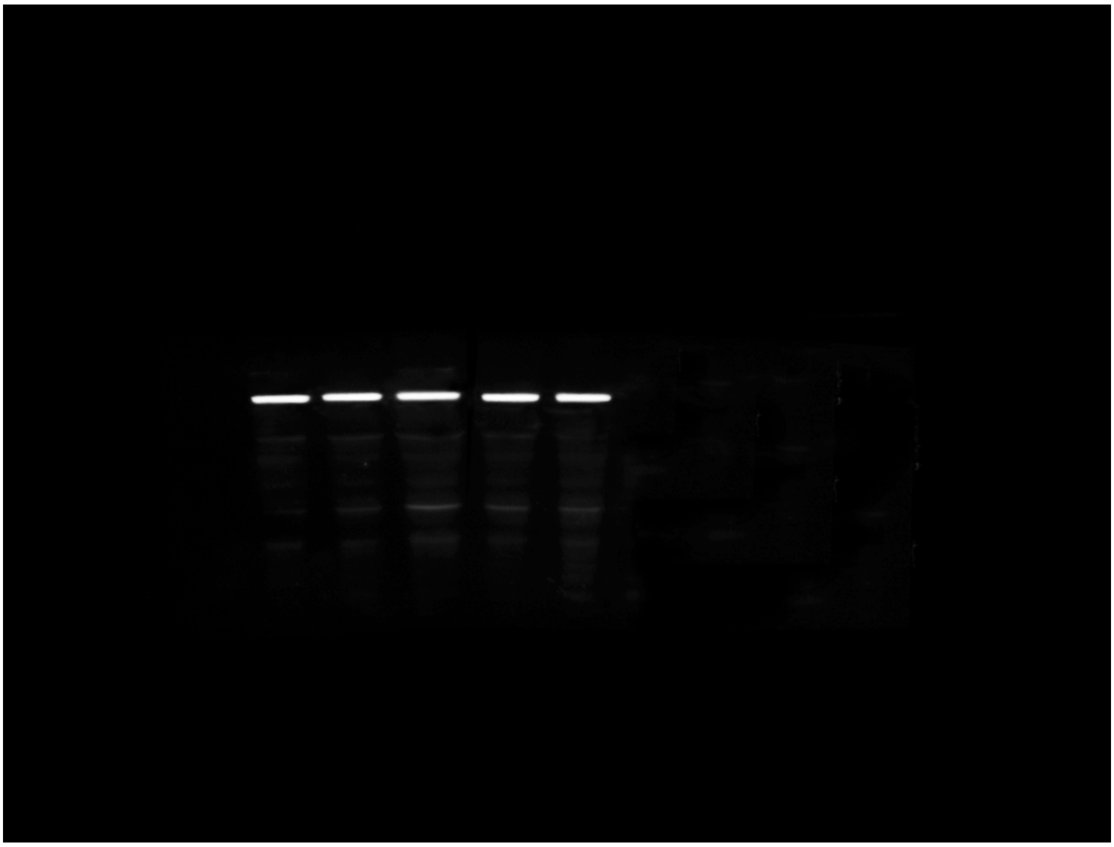

LPA

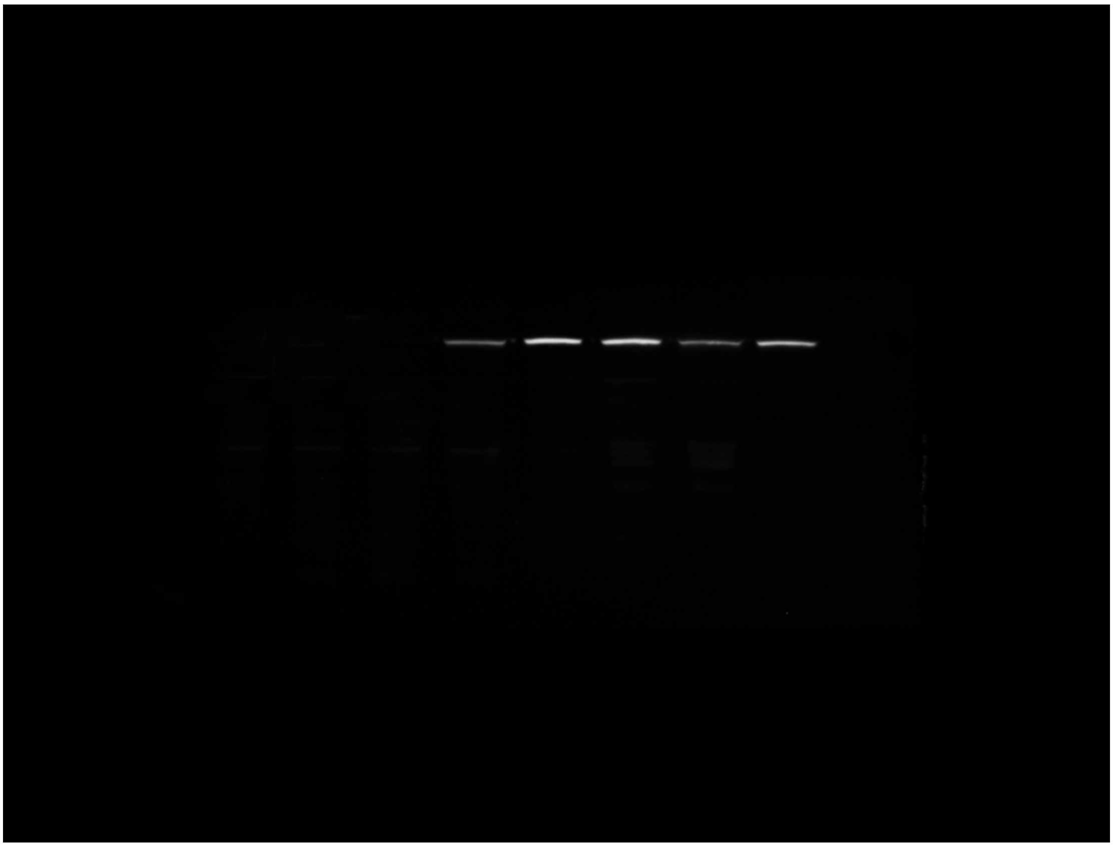

UCP1

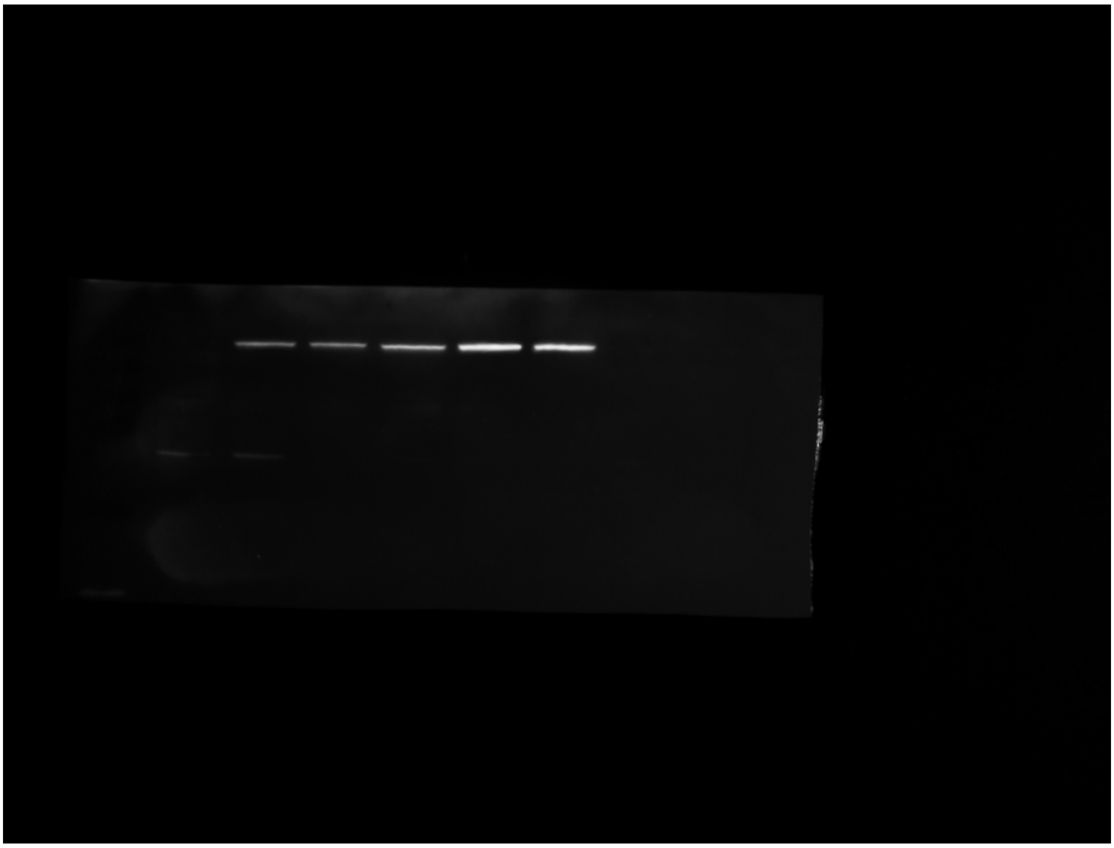

ITGAM

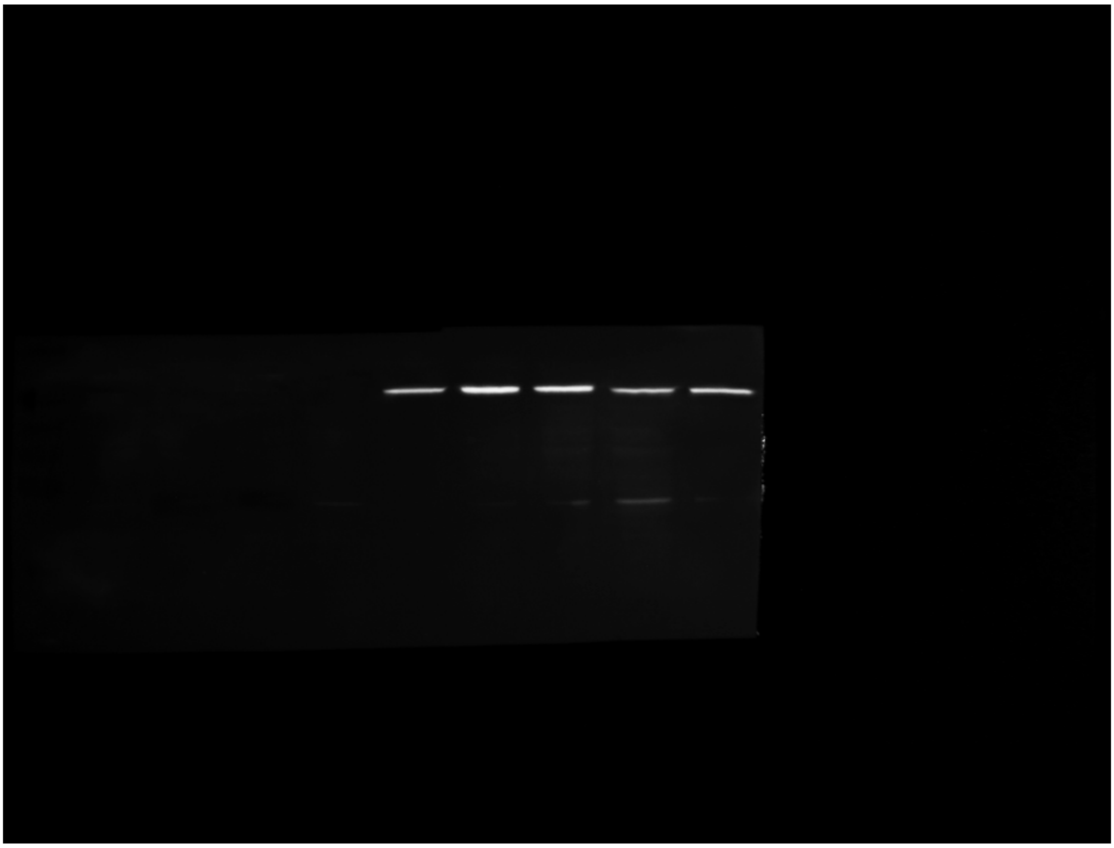

NF-κB

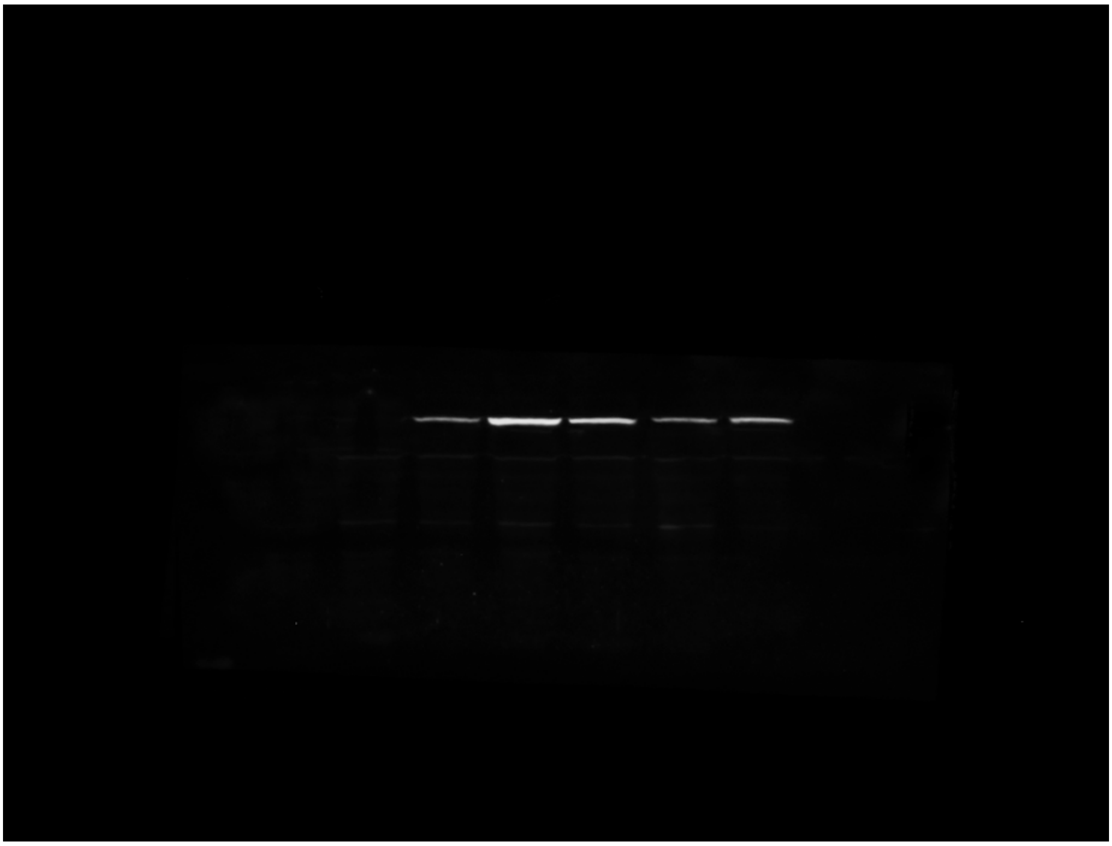

Supplement: Supplementary Materials — Supplementary file WB of Figure 5A: antibodies for western blot are as follows: anti-PPAR-γ (catalog: 95128S, CST), anti-FABP4 (catalog: 2120, CST), anti-LPL (catalog: ab137821, abcam), anti-UCP1 (catalog: 14670, CST). The groups from left to right are White adipocyte and Brown adipocyte. Supplementary file WB of Figure 5C: antibodies for western blot are as follows: anti-LPA (catalog: ab33470, abcam), anti-UCP1 (catalog: 14670, CST), anti-ITGAM (catalog: 46512, CST), anti-NF-κB (catalog: 6956, CST). The groups from left to right are NC, Model, Model+Adipocyte+shPPARγ, Model+Adipocyte+rosiglitazone, and Model+Adipocyte. [file 1731540.f1.pdf]
